# Plant-Based Nutrition: Exploring Health Benefits for Atherosclerosis, Chronic Diseases, and Metabolic Syndrome—A Comprehensive Review

**DOI:** 10.3390/nu15143244

**Published:** 2023-07-21

**Authors:** Humberto Peña-Jorquera, Valeska Cid-Jofré, Leslie Landaeta-Díaz, Fanny Petermann-Rocha, Miquel Martorell, Hermann Zbinden-Foncea, Gerson Ferrari, Carlos Jorquera-Aguilera, Carlos Cristi-Montero

**Affiliations:** 1 IRyS Group, Physical Education School, Pontificia Universidad Católica de Valparaíso, Viña del Mar 2530388, Chile; humberto.apj@gmail.com; 2 Centro de Investigación Biomédica y Aplicada (CIBAP), Escuela de Medicina, Facultad de Ciencias Médicas, Universidad de Santiago de Chile (USACH), Santiago 9160019, Chile; valeska.cid@usach.cl; 3 Facultad de Salud y Ciencias Sociales, Universidad de las Américas, Santiago 7500975, Chile; llandaeta@udla.cl; 4 Núcleo en Ciencias Ambientales y Alimentarias, Universidad de las Américas, Santiago 7500975, Chile; 5 Centro de Investigación Biomédica, Facultad de Medicina, Universidad Diego Portales, Santiago 8370068, Chile; fanny.petermann@udp.cl; 6 BHF Glasgow Cardiovascular Research Centre, School of Cardiovascular and Metabolic Health, University of Glasgow, Glasgow G12 8TA, UK; 7 Department of Nutrition and Dietetics, Faculty of Pharmacy, Centre for Healthy Living, University of Concepción, Concepción 4070386, Chile; mmartorell@udec.cl; 8 Laboratorio de Fisiología del Ejercicio y Metabolismo, Escuela de Kinesiología, Facultad de Medicina, Universidad Finis Terrae, Santiago 7500000, Chile; hzbinden@uft.cl; 9 Facultad de Ciencias de la Salud, Universidad Francisco de Vitoria, 28223 Madrid, Spain; 10 Facultad de Ciencias de la Salud, Universidad Autónoma de Chile, Av. Pedro de Valdivia 425, Providencia 7500912, Chile; gerson.demoraes@usach.cl; 11 Escuela de Ciencias de la Actividad Física, el Deporte y la Salud, Universidad de Santiago de Chile (USACH), Santiago 9170022, Chile; 12 Escuela de Nutrición y Dietética, Facultad de Ciencias, Universidad Mayor, Santiago 8580745, Chile; carlos.jorquera@mayor.cl

**Keywords:** vegetarian diet, plant bioactive compounds, cholesterol, hyperinsulinemia, blood pressure

## Abstract

Atherosclerosis, chronic non-communicable diseases, and metabolic syndrome are highly interconnected and collectively contribute to global health concerns that reduce life expectancy and quality of life. These conditions arise from multiple risk factors, including inflammation, insulin resistance, impaired blood lipid profile, endothelial dysfunction, and increased cardiovascular risk. Adopting a plant-based diet has gained popularity as a viable alternative to promote health and mitigate the incidence of, and risk factors associated with, these three health conditions. Understanding the potential benefits of a plant-based diet for human health is crucial, particularly in the face of the rising prevalence of chronic diseases like diabetes, hypertension, dyslipidemia, atherosclerosis, and cancer. Thus, this review focused on the plausible advantages of consuming a type of food pattern for the prevention and/or treatment of chronic diseases, emphasizing the dietary aspects that contribute to these conditions and the evidence supporting the benefits of a plant-based diet for human health. To facilitate a more in-depth analysis, we present separate evidence for each of these three concepts, acknowledging their intrinsic connection while providing a specific focus on each one. This review underscores the potential of a plant-based diet to target the underlying causes of these chronic diseases and enhance health outcomes for individuals and populations.

## 1. Introduction

Many people consider a plant-based diet (PBD), which includes only plant sources with the absence or occasionally minimal presence of processed food, a novel and even risky eating choice. This concern is heightened by the potential deficiencies in micronutrients, such as vitamin B12 and D, calcium, omega 3, or iron, compared to a traditional diet [1,2]. However, over the past decades, a wealth of scientific evidence has accumulated that provides strong support for the potential health benefits of a PBD. These benefits include the prevention of various chronic non-communicable diseases, such as type 2 diabetes [3,4], hypertension [5,6,7], dyslipidemia [8,9], atherosclerosis, and cancer [10,11]. Studies on supplementation and natural consumption approaches have demonstrated the practical advantages of a PBD, which are attributed to the bioactive compounds present in plants, such as catechins [12,13], anthocyanins [14,15], polyphenols [16], and phytosterols [17,18], among others.

Metabolic syndrome (MetS) is associated with several adverse effects on human health. Although there are various definitions, components, and criteria for MetS, they all include visceral obesity, insulin resistance, hypertension, and dyslipidemia [19]. According to National Cholesterol Educations Program Adult Treatment Panel ATP III, MetS is considered present when an individual meets at least three of the following five criteria: waist circumference over 40 inches (men, or >102 cm) or 35 inches (women, or >88 cm), blood pressure over 130/85 mmHg, fasting triglyceride level over 150 mg/dL, fasting HDL cholesterol level less than 40 mg/dL (men) or 50 mg/dL (women), and fasting blood sugar over 100 mg/dL [20]. Other entities even consider more demanding cut-off points [21]. Individuals with MetS have higher cardiovascular disease and all-cause mortality risk compared to whose without MetS [22,23].

In this line, a PBD has been found to have numerous positive and protective effects on metabolic health, significantly reducing the associated risks. For instance, Jovanovic et al. [24] concluded that a 1-unit increase in daily servings of a healthy plant-based diet, which excludes added sugars, refined grains, and oils, was associated with a 4% lower risk prevalence of elevated waist circumference and MetS risk. However, not all evidence supports these outcomes. Shang et al. [25] found that a vegan diet alone did not decrease the risk of MetS, but the study only assessed the absence of animal foods (meat, dairy, and eggs), not the quality of the diet. Previous research suggests that adherence to a healthful plant-based diet, which includes increased fiber intake, plant bioactive compounds, and lower consumption of ultra-processed foods, is associated with benefits related to MetS [26,27]. It is worth noting that there is a vast difference in quality and health outcomes between a healthy and an unhealthy PBD. Indeed, Li et al. [28] in 2022 established that a healthy PBD is associated with lower mortality risk than an unhealthy PBD.

A vegan diet is often used as an equivalent to a PBD, although considerable differences exist between these two concepts, both nutritionally and ethically [29]. While the first is exclusively related to selecting food for ethical reasons (animal empathy), the second is related to health and/or environmental protection. Additionally, a vegan diet does not necessarily prioritize food quality. In contrast, a PBD emphasizes consuming whole foods and minimally processed products, focusing on legumes, whole grains, fruits, vegetables, seeds, and nuts [30].

As defined by some authors, plant-based eating patterns include fish, poultry, and yogurt [31,32,33]. However, this definition is more accurately described as a pescatarian diet, including other seafood, or as a lacto-vegetarian diet. Other authors have included a low frequency of animal sources as a definition of a PBD [34], while other sources explicitly highlight that a PBD does not necessarily mean being vegetarian or vegan [30]. It is central to recognize these differences to fully understand the potential benefits and limitations of a PBD and make informed dietary choices.

This review aimed to highlight the benefits of diverse plant bioactive compounds and emphasize the significance of including plant-origin macronutrients, vitamins, and minerals in our diets to prevent and/or treat the pathogenesis of chronic non-communicable diseases, metabolic syndrome indicators, and atherosclerosis due to increased prevalence and incidence globally.

## 2. Plant-Based Diet and Atherosclerosis

### 2.1. Brief Summary of the Pathophysiology and Confounding Outcomes

**Figure 1:**
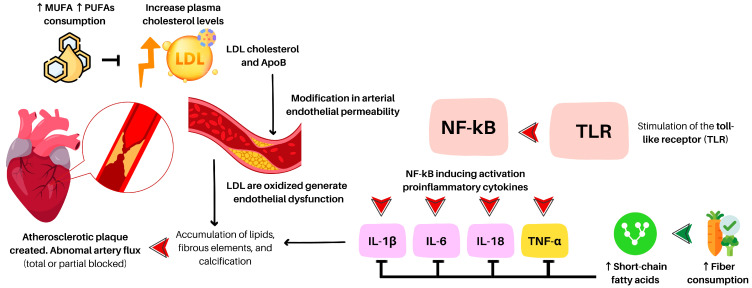
A brief description of the atherosclerosis process. MUFA: monounsaturated fatty acids; PUFA: polyunsaturated fatty acids; LDL: low-density lipoprotein; Apo-B: apolipoprotein B; NF-kB: nuclear factor-κB; IL: interleukin; TNF-α: tumor necrosis factor alpha.

Atherosclerosis (ATE) is considered a principal cause of different coronary heart diseases. It is characterized by the accumulation of lipids, fibrous elements, and calcification within the large arteries, similar to a chronic inflammatory process [35]. This involves the stimulation of the toll-like receptor (TLR), which activates the transcription factor (nuclear factor-kappa beta), inducing the activation of proinflammatory components such as interleukin 1β (IL-1β), IL-6, IL-18, and tumor necrosis factor-alpha (TNF-α) [36] (see Figure 1).

An increase in plasma cholesterol levels can result in changes in arterial endothelial permeability, leading to the migration of lipids, particularly low-density lipoprotein cholesterol particles, into the arterial wall [37]. This process can be upregulated by certain proinflammatory conditions such as advanced glycation end-products (AGEs) [38], hypercholesterolemia, hypertension [39], or type 1 diabetes [40]. The trapped lipoproteins are oxidized, leading to endothelial dysfunction [36] and forming foam cells. LDL particles transport different components, including apolipoprotein B100 (Apo-B100) [41]. Recent evidence [42,43] suggests that Apo-B content is linked to ATE as the primary cause of atherogenic pathology.

Previous studies have identified that Apo-B, not LDL cholesterol, is strongly associated with coronary artery calcification [44,45]. However, a recent Mendelian randomized analysis showed that, in individuals with equal levels of non-HDL cholesterol, the development of coronary artery disease is not influenced by the number of Apo-B particles carried, suggesting that the clinical impact of lipid-lowering therapies is expected to be proportional to the reduction in non-HDL cholesterol rather than the reduction in Apo-B [46]. Thus, the LDL particle oxidation process is also a critical factor that should be considered, as it has been strongly linked to coronary atherosclerosis, arterial dysfunction, and mortality, affecting elasticity and vasodilatory endothelial vascular function [47,48]. In this line, it has been suggested that the regulation of oxidative stress could be one of the major strategies to reduce the trapping of Apo-B in the intima, decreasing atheroma formation, inflammation, and atherosclerosis pathogenesis [36].

In parallel, it has been established that people who do not develop atherosclerosis have an optimal and normal LDL cholesterol range of 50–70 mg/dL [49]. In a Consensus Statement from the European Atherosclerosis Society Consensus Panel (EAS), Ference et al. [50] emphasized the consistent evidence from clinical and genetic studies that unequivocally establishes the role of LDL in causing atherosclerotic cardiovascular disease (ASCVD). Despite that, not all the evidence supported this statement [51], which could be explained by reverse causation [52,53]. While observational studies in middle-aged individuals have reported a positive association between cardiovascular disease and cholesterol levels, the role of high cholesterol as a cardiovascular risk factor in individuals above 75 years old is controversial [54].

Overall, LDL cholesterol levels in plasma may not reflect lifetime LDL cholesterol levels due to comorbidities [55]. To address this issue, the authors of the study mentioned above [54] used LDL-GRS (genetic risk score) and found that the genetic predisposition to high LDL cholesterol levels contributes to mortality throughout life, including in the oldest individuals. Finally, the coexistence of coronary artery disease and malnutrition may reflect the intriguing phenomenon known as the “cholesterol paradox.” The last concept refers to a disparity in some of the results found and what the literature explains. In this line, a previous study [56] concluded that the worse mortality prognosis observed in patients with low LDL cholesterol group (<1.8 mmol/L) is mainly mediated by their higher prevalence of malnutrition. After adjustment for malnutrition, patients with coronary artery disease who had low baseline serum LDL cholesterol concentrations had a low risk of long-term all-cause mortality.

### 2.2. Saturated and Unsaturated Fat

A diet high in sugar, salt, cholesterol, and fat—commonly called a Western diet—has been linked to various health issues, including diabetes mellitus, high blood pressure, hyperlipidemia, obesity, and coronary artery disease. These conditions can promote atherogenesis, atherosclerosis, and atherothrombotic coronary artery disease [57]. However, a recent animal study by Huang et al. [58] showed that the adverse effects of Western diet-induced atherosclerosis could be mitigated by down-regulating obesity, inflammation, and chemotaxis signaling. These factors are modulated by the microbiota and derived short-chain fatty acids (SCFAs).

In contrast, a diet rich in extra-virgin oil and nuts has been shown to have beneficial effects. Compared to a Western diet, a diet high in unsaturated fats can lead to lower plasma cholesterol and triglyceride levels, as well as reduced inflammation and atherosclerosis in animal models (inhibited foamy monocyte formation, inflammation, adhesion, and reduced atherosclerosis in Ldlr -/- mice) [59]. Previous human research has shown that a high-unsaturated fat diet and a very low-fat diet can lead to a more significant decrease in LDL cholesterol than a high-saturated fat diet [60]. Increasing poly and mono-unsaturated fatty acids (PUFAs and MUFAs, respectively) reduce cardiovascular disease events mainly due to the degree of cholesterol-lowering. The cardiovascular effects of reducing saturated fat rely on changes in atherosclerosis via serum cholesterol [61], influencing pathways affecting inflammation, cardiac rhythm homeostasis, apolipoprotein-C III production, and high-density lipoprotein (HDL) function [62].

Notwithstanding the above, some authors [63] consider that American guidelines and recommendations may be biased and that saturated fat in certain foods, such as whole fat dairy or dark chocolate, can benefit health and are not associated with cardiovascular disease or diabetes. While Gershuni [64] supported this general conclusion, he also indicated that the saturated fatty acid found in meat, eggs, cacao, and nuts is primarily composed of triglycerides containing palmitic acid and stearic acid, covering 90% of fatty acid in the standard American diet. However, exogenous palmitic acid can exert a different effect depending on the source. In both animal and human in vivo and in vitro studies [65,66], palmitic acid has been associated with promoting atherosclerosis development due to cholesterol accumulation in LDL particles and macrophages activating the inflammatory process [67]. Further, elevated palmitic acid levels enhanced the uptake of oxidized LDL via the upregulation of lectin-like oxidized LDL receptors in macrophages, mediated by ROS-p38 pathways rather than TLRs [68].

A vast study involving 76,364 women observed that the consumption of high-fat foods such as peanuts and tree nuts (two or more times a week) and walnuts (one or more times a week) was associated with a 13–19% lower risk of total cardiovascular risk disease and a 15–23% lower risk of coronary heart disease [69,70]. In an animal study [71], a high-fat diet rich in walnuts was found to cause a 55% reduction in atherosclerotic plaque development in the aortic arch compared to the control diet. Urpi-Sarda et al. [72] found that a Mediterranean diet with virgin olive oils and nuts can down-regulate cellular inflammatory biomarkers associated with atherogenesis [73] and modify the process of the firm adhesion of circulating monocytes and lymphocytes T to endothelial cells during inflammation [74]. These findings suggest that adding nuts to a Mediterranean diet or adopting a whole-food vegan diet can reverse the atherosclerotic process of coronary artery disease [75].

In this line, a meta-analysis [76] demonstrated that replacing 1% of the dietary carbohydrate with MUFAs or PUFAs resulted in increased HDL cholesterol, decreased triacylglycerol concentration, and attenuated increases in LDL and total cholesterol levels. In contrast, a study on coconut oil found that reducing saturated fat without changing the polyunsaturated/saturated fatty acid ratio (P/S) did not lower total or LDL cholesterol but significantly reduced HDL cholesterol. However, a diet high in MUFAs and PUFAs resulted in a greater reduction in LDL cholesterol, lower LDL/HDL cholesterol, and an improved Apo-B/Apo-A ratio [77]. This last conclusion is fundamental, considering LDL/HDL ratio is suggested as a sensitive predictor of coronary atherosclerotic heart disease (CADH) [78]. Overall, although nutritional evidence has not convincingly shown that plant-based fats alone significantly improve HDL cholesterol, there is evidence that they can lower LDL cholesterol and maintain unchanged HDL cholesterol, thus improving the LDL/HDL ratio [79,80].

### 2.3. Trimethylamine N-Oxide and Gut Microbiota

Trimethylamine N-oxide (TMAO) is a compound that has gained interest due to its potential mechanistic links to atherosclerosis heart disease [81]. Trimethylamine (TMA) is generated by gut microbiota in response to nutrients, with eggs and meat being major dietary sources of the TMA precursor. In the liver, TMA is transformed into TMAO by flavin-containing monooxygenase 3 [82]. While a considerable body of evidence strongly supports the detrimental impact of TMAO on health, some results are inconsistent, with stronger relations observed in patients with preexisting medical conditions compared to healthy subjects [83]. However, in a previous Mendelian randomization analysis [84], the authors found that some chronic non-communicable diseases like type 2 diabetes mellitus and kidney disease increase TMAO levels, and such observational evidence for cardiovascular disease may be due to confounding or reverse causality. Despite the above, different non-modifiable factors increase plasma TMAO levels, such as age, sex, and genetic factors. However, various components found in animal and plant food can also potentially increase it [83]. Choline, phosphatidylcholine, l-carnitine, betaine, crono-betaine, and γ-butyrobetaine [85,86] can be used as precursors by gut microbiota to generate TMAO.

Kühn et al. [87] established that TMAO levels could be more affected by intra-individual variation, which could mediate the result due to physical activity or intestinal microbiota. Depending on the context, these factors can positively or negatively modify the type of intestinal bacteria present. For instance, betaine, mainly found in plants, can potentially affect TMAO levels and be synthesized from dietary choline. Betaine also serves as an osmoprotectant in the kidney and plays a crucial role in the methionine-homocysteine cycle, maintaining the s-adenosylmethionine/s-adenosyl-homocysteine ratio in the liver, especially when folate is insufficient [88]. However, some studies have shown no relationship between dietary betaine and the incidence of cardiovascular disease, even though TMAO increased cardiovascular mortality in some populations [89,90]. A possible explanation lies in the composition of the gut microbiota.

In a previous study [82], a carnitine challenge test was conducted on omnivorous and vegetarian/vegan participants using steak and/or veggie caps containing 250 mg of a stable isotope-labeled d3-L-carnitine to measure TMAO levels. The results showed that vegetarians/vegans challenged with d3-carnitine had a significantly reduced synthetic capacity to produce TMAO from oral carnitine compared to omnivorous individuals. Some authors have suggested that the richness, expressed in the number of species, of the intestinal microbiota may impact the host’s health, although this is still subject to debate [91]. 

However, De Filippo et al. [92] conducted previous research comparing the human intestinal microbiota among children from Burkina Faso (rural context) with a diet low in fat and animal protein, rich in starch, fiber, and plant polysaccharides, legumes, sorghum, and millet grain being predominantly vegetarian, versus children from Italy (urban context) with a diet high in animal protein, sugar, starch, fat, and low in fiber. The authors found that diet plays a primary role influencing the composition and diversity of the microbiota, suggesting that diet has a dominant influence over other variables such as hygiene, sanitation, ethnicity, geography, and climate. Gut bacteria can generate SCFA byproduct formation, named butyrate, propionate, and acetate, which help maintain normal large bowel function, prevent pathologies through their influence in the gut lumen, the colonic musculature and vasculature and through their metabolism by colonocytes [93]. At the same time, increased SCFA from plant-based sources has been associated with reduced TMAO levels and atherosclerotic risk.

Concerning protein consumption, an early culture-based study on gut microbiota [94] demonstrated lower counts of *Bifidobacterium adolescentis* and increased counts of Bacteroides and Clostridia in subjects consuming a high beef diet compared to those on a meatless diet. In a more recent study, Singh et al. [95] analyzed the effect of different types of protein (animal-based protein and plant-based protein) and found that pea protein increased intestinal SCFAs levels, which are considered anti-inflammatory and essential to the maintenance of the mucosal barrier [96]. This increase in SCFAs was associated with an increased prostaglandin E1/prostaglandin E2 ratio produced by subepithelial myofibroblasts enhancing mucin-2 (MUC-2) expression in epithelial cells [97]. These positive effects on the gut barrier, especially in MUC-2, help to reduce bacterial translocation (endotoxemia), gut permeability, inflammation, and TMAO levels [98,99,100,101].

Contrary to what has been stated, recent studies have shown that TMAO levels are positively correlated with the intake of vegetables and whole-grain cereal, contradicting the notion that a healthy diet may help reduce TMAO levels [102]. Similarly, Griffin et al. [103] found that a Mediterranean diet intervention over six months did not significantly mitigate TMAO concentration in a healthy population. These findings call into question the effectiveness of a high plant-based diet in reducing TMAO levels and, consequently, the risk of cardiovascular and coronary heart disease.

### 2.4. Plant-Based Diet, LDL Cholesterol, TMAO, and Atherosclerotic Risk

In a recent randomized cross-over study [104], the authors observed that a PBD that includes whole eggs might maintain or improve dyslipidemia, oxidative stress, and inflammation biomarkers over vegan or lactovegetarian diets in individuals with MetS. The study suggested that egg consumption may have theoretical benefits, such as increasing HDL cholesterol, without causing any adverse effects on LDL cholesterol, triglycerides, or glucose levels. These findings were consistent with previous research by Zhu et al. [105], who found that two eggs/day in overweight postmenopausal women significantly increased plasma choline and betaine levels but did not alter TMAO levels or gut microbiome composition.

In a previous study (2005) related to this topic [106], it was established that individuals over 60 years old with a healthy lipoprotein profile might consume eggs as part of their regular diet due to eggs consumption possibly increasing LDL cholesterol, but an increase in HDL cholesterol offsets this elevation. However, a meta-analysis [107] conducted earlier (2001) indicated that the beneficial rise in HDL cholesterol by consuming eggs is insufficient to offset the negative rise in total LDL cholesterol concentrations, implying that an increase in dietary cholesterol intake may increase the risk of coronary heart disease. Further, several decades ago, some studies [108,109,110] clearly and unequivocally established that egg consumption leads to an increase in total and LDL cholesterol, although the extent of the increase depends on baseline cholesterol levels [111]. However, and related to TMAO, a more recent study [112] concluded that egg consumption did not increase its levels of plasma. Notwithstanding, in this study, the authors used a 12-h fasting protocol to measure TMAO levels, and previous evidence has indicated that the kidneys efficiently eliminate TMAO to maintain a steady state of circulating choline levels in a couple of hours [113,114].

### 2.5. Endothelial Vascular Function

Endothelial vascular dysfunction is among the risk factors involved in coronary artery disease [57]. High salt consumption has presented strong evidence of the adverse effect on endothelial function [115,116] by stiffening human endothelial cells and reducing nitric oxide (NO) production [115]. NO is a key signaling molecule that regulates blood flow and tissue oxygenation, and its bioavailability reduction augments atherosclerosis risk [117], associated with an impairment of endothelium-dependent relaxation. In addition, a single high-fat (50 g) meal reduces blood flow-mediated vasoactivity in the 2- to 4-h postprandial period [118]. This previous outcome has also been reported by Keogh et al. [119], who detailed that a high-saturated fat diet causes deterioration in flow-mediated dilation (FMD) compared with a high PUFA, MUFA, or even CARB (carbohydrate) diet.

Contrary to what has been previously described, a singular food and/or full PBD has been recognized as a healthy eating pattern by improving FMD. An 8-week cross-over feeding trial demonstrated that walnuts consumption as a substitute for 32% of the energy from MUFAs in a cholesterol-lowering Mediterranean diet improves vascular endothelial function [120]. A hazelnut-enriched diet for four weeks has also shown a significantly improved FMD in hypercholesterolemic subjects, besides improving TC (total cholesterol), TG (triglycerides), LDL, and HDL as well oxidized LDL, CRP (c-reactive protein), and soluble vascular cell adhesion molecule-1 compared with the control diet [121].

Another study found that daily walnut consumption (56 g) improves endothelial function in overweight adults with visceral adiposity [122]. Daily consumption of high-flavanol cocoa drinks has been shown to lead to a sustained reversal of endothelial dysfunction in approximately five days. Interestedly, the magnitude of this positive effect observed with a high-flavanol cocoa drink was similar to that observed in a long-term pharmacological approach with statins [123,124]. Another study using daily inorganic nitrate as beetroot juice for six weeks in hypercholesterolemic individuals showed a ∼24% improvement in FMD through the rising in nitrate circulation. This beneficial effect was related to a reduction in platelet-monocyte aggregate numbers and reduced platelet P-selectin expression [125], associated with cardiovascular disease progression, which can initiate the release of atherogenic proinflammatory and adhesive molecules and induce procoagulant microparticle formation, respectively [126,127].

In this context, nitrate and nitrite have been widely recognized as nitric oxide precursors. In the case of nitrate, certain plant foods can improve vascular function through this content and enhance NO formation. Spinach, watercress, chervil, chard, arugula, beets, celery, and lettuce are some plant foods with high nitrate concentrations [128]. In this line, Bondonno et al. [129], in a study of older women, observed an inverse association between the intake of vegetable nitrates with CCA-IMT (intima-media thickness of the common carotid artery) and the risk of an ischemic cerebrovascular event for 15 years. This result was not observed with non-vegetable nitrates consumption. Although some evidence has demonstrated an insignificant change in a single and specific indicator, such as systolic blood pressure, after five weeks of supplementation of leafy green vegetables or pills containing the same amount of inorganic nitrate [130], most of the literature corroborates the benefits of PBD for the improvement of vascular function.

Prolonged consumption of soy nuts as part of a healthy diet improves endothelial function, LDL cholesterol concentration, and mean arterial pressure [131]. In this recent study, the enhancement is more related to restoring a condition of nitric oxide impairment rather than enhancing a normal physiological condition [132]. In contrast, previous evidence has shown that fish, with green tea and a lower consumption of saturated fat as part of a traditional Chinese diet, also improves endothelial function in older Chinese people’s arteries [133]. However, in later studies, fish oil supplementation or whole-fish consumption showed no significant effect on endothelial function [134,135,136]. Additionally, in a comparative study between lacto-ovo-vegetarians and omnivores on the measurement of vascular dilator function, the authors found that the vegetarian diet, by itself, has a direct beneficial effect on the vascular endothelium and the function of the smooth muscle, and may help explain the lower incidence of atherosclerosis and cardiovascular mortality [137].

### 2.6. Short-Chain Fatty Acids, Gut Microbiota, and Atheroma Formation

Gut microbiota, mainly through probiotics, bioactive compounds intake, and SCFA formation, exerts multiple health benefits, having a significantly positive role in the reduction of atherosclerosis. Soybeans, such as tempeh, are rich in bioactive compounds like genistein and daidzein. These isoflavones are present in different legumes but are markedly higher in soybean. The growing body of evidence around isoflavones in the last 15 years suggests their beneficial effects on preventing breast and prostate cancer, cardiovascular disease, and chronic non-communicable diseases. In animal models, type 2 diabetes has been demonstrated to aggravate colonic damage and inflammation response and decrease levels of SCFA [4] leading to dysbiosis. Gut dysbiosis can alter different homeostatic functions increasing the pathophysiology risk of several complications like diabetes or atherosclerosis, among others [138,139].

Previous evidence has observed different compositions of bacteria between patients with or without symptomatic atherosclerosis [140]. The production of SCFAs can inhibit foam cell formation by stimulating the expression of IL-10 and reducing the production of proinflammatory cytokines by the endothelium, contributing to the recovery of endothelial dysfunction and reducing atherosclerotic risk [141].

Inflammation can promote alteration in the gut barrier. Increasing gut permeability is associated with inflammation and reduced expression of specific tight junction proteins, such as zonula occludens-1, claudin-1, and occluding [142,143,144]. The reduction of its expression increases bacteria translocation of LPS (lipopolysaccharides) and proinflammatory cytokines. Therefore, SCFAs can reduce gut permeability by decreasing nuclear factor-kappa B (NF-kB) activation and, consequently, reduce proinflammatory cytokines such as IL-1b, IL-6, IL-8, and TNF-α [145]. Additionally, SCFA has proved to attenuate NF-kB and peroxisome proliferator-activated receptors’ (PPARγ) activities and, consequently, suppress adhesion molecules expression like vascular cell adhesion molecule-1 and intercellular adhesion molecule-1 [146]. Accompanying these effects, they showed increased anti-inflammatory actions. For instance, in macrophages, butyrate had anti-inflammatory effects by decreasing inducible nitric oxide synthase, TNF-α, monocyte chemo-attractant protein-1, and IL-6 production through the activation of free fatty acids 3 receptors, which has been implicated in obesity and metabolic diseases [147]. In a mouse study [148], genistein has been shown to increase SCFAs concentration and modulate gut microbiota in mice.

In a regular omnivorous diet, the average daily fiber consumption is lower than recommended [149]. Additionally, Davies et al. [150] observed that omnivorous, vegetarian, and vegans reported different fiber intakes, with 23, 37, and 47 g/d, respectively. In both genders, the highest versus lowest fiber intake was associated with a 22% decrease in mortality [151] and reduced long-term ASCVD [152]. Nowadays, fiber is known as a microbiota-accessible carbohydrate (MAC) and represents the primary energy source for colonic bacteria. When fiber intake reaches 50–120 g/day, it is associated with a more diverse gut microbiota than people in Western countries. In the latter, it has been correlated with highly prevalent diseases [153].

Even so, the connection between MAC and TMAO formation is still debatable. Some authors propose that a high non-digestible carbohydrate diet may reduce TMAO formation by modulating the gut microbiota, but conflicting findings have been reported [154]. However, a previous study to determine the impact of fiber deprivation over four generations on the gut microbiota in mice colonized with human microbiota from a Westerner diet showed that the consumption of a regular Western low-fiber diet contributes to the loss of taxa over generations and may be responsible for the lower diversity of microbiota observed. The re-introduction of dietary MAC was insufficient to recover taxa [155].

In the context of human health, consuming plant-based foods promotes the development of a more diverse gut microbial community and may also impact the distribution of different species within it [156]. The difference in gut microbiota composition between omnivorous and vegetarians/vegans has been well documented, and fiber consumption has shown an inverse relationship with cardiovascular disease, including atherosclerosis [152,157]. A soy-based diet has been shown to reduce the risk of atherosclerosis by inhibiting the formation of foam cells in macrophages. Downregulating scavenger receptors achieve this effect in a cell culture model using THP-1 macrophages, which is attributed to the presence of soy pinitol, which could inhibit oxidized LDL formation [158]. Later, evaluating the consumption of a single high-fat meal, this study found that a high-fat meal resulted in a transient increase in acLDL (acetyl low-density lipoprotein) endocytosis and adhesion molecule expression in both classical and nonclassical monocytes, increasing the susceptibility to foam cell formation [159]. However, this study did not clarify the specific content of every meal, which varied between subjects. Therefore, it is essential to differentiate the effects of different diets and fat sources.

### 2.7. Fermented Plant-Food and Atherosclerosis

Rabbits subjected to a high-cholesterol diet experienced significant health complications. The progression was retarded by the administration of 3-(4’-hydroxyl-3’,5’-dimethoxyphenyl) propionic acid (HDMPPA), an active compound of kimchi, which can suppress TC and LDL cholesterol elevation, reducing the thickness of the aortic arch and antioxidant activity [160]. Yun et al. (2014) [161] also found that HDMPPA had a protective effect on the cell viability of THP-1-derived macrophages through the inhibition of lipid peroxidation, regulating cluster of differentiation 36 and ABCA1 expression, both of which at least partially participate in cholesterol influx and efflux. This context could regulate foam cell formation by attenuating cholesterol accumulation in macrophages.

Additionally, it is well documented that whole grains can improve health in different contexts. For instance, a 12-week whole-grain wheat-based diet increases fasting plasma propionate, a type of SCFA, in individuals with metabolic syndrome [162]. Interestingly, Lisosan G (LG), a fermented powder obtained from whole grain, has demonstrated an antioxidant and anti-inflammatory capacity [163]. Moreover, LG protects EPCs exposed to LPS, reducing intracellular reactive oxygen species (ROS), and is capable of inducing vascular damage and lowering or normalizing cytokine [164].

A study found that fermented plant beverages, in this case kombucha with pollen, led to a significant increase in SCFAs, probably depending on a mixture of microorganisms called the symbiotic culture of bacteria and yeast (SCOBY) [165]. In another beverage study, fermented Korean tea (chungtaejeon) was proven to scavenge oxidation and inhibit the cytokine-induced proliferation and migration of human aortic smooth muscle cells. Vascular smooth muscle cells secrete TNF-α, activating ERK1/2, a crucial mediator of signals that promote cell growth and motility. This pathway plays a pivotal role in the development of vascular lesions. Also, the chungtaejeon beverage inhibited the enzymatic action and protein expression of TNF-α-induce matrix-metalloproteinase (facilitates migration of vascular smooth muscle cells via matrix disruption contributing to the pathogenesis of atherosclerosis) in human aortic smooth muscle cells [166].

Moreover, red yeast rice has been reported to confer multiple health improvements, including atherosclerosis and lipid profile. Monacolin K is believed to be the key factor responsible for these positive effects, as recently reported by Rahmani et al. [167]. In an 8-week intervention study, a daily dose of 200 mg red yeast rice containing 2 mg of monacolin K significantly reduced LDL cholesterol, blood pressure, and Apo-B levels compared to the control group [168]. The mechanisms involved in these modifications are generated through various pathways, including cholesterol biosynthesis, LDL receptor metabolism, inhibiting acyl-coenzyme A-cholesterol acyltransferase, decreasing the conversion of cholesterol-to-cholesterol esters and the secretion of Apo-B, enhancing endothelial cell function due to NO activity, reducing ROS, preventing a connection between Lox-1 and ox-LDL, and reducing proinflammatory cytokines, among others [169].

### 2.8. Bioactive Compounds

Plant-derived bioactive components have auspicious therapeutic attributes, especially antioxidative properties [170]. Thus, both independently and in combination with other bioactive compounds found in plant foods, they have been shown to contribute to reducing plasma cholesterol. For instance, a pilot study [171] using supplemented pasta with 6% of β-glucan showed, after 30 days, a significant reduction in LDL cholesterol, IL-6, AGEs levels, and oxidative stress. In addition, it has been suggested by other researchers [172] that β-glucan exhibits significant physicochemical properties, including its antioxidant capability to scavenge reactive oxygen species, its role as a dietary fiber to inhibit cholesterol absorption, and its ability to promote the production of short-chain fatty acids (SCFAs). A previous meta-analysis has supported most of these results, indicating that barley β-glucan can significantly lower LDL and non-HDL cholesterol [173]. In another study, it was observed that a daily intake of 3 g of oat β-glucan safely reduced total, LDL, and non-HDL cholesterol in a large cohort of adults with mild hypercholesterolemia and a low cardiovascular risk profile [174].

In addition, berries as whole fruits, juice, or extract, decrease plasma LDL cholesterol and triglycerides and/or increase HDL cholesterol in individuals who exhibit elevated lipid biomarkers [175]. The modulating lipid metabolism primarily involves increasing the hepatic synthesis of apolipoprotein A-I, downregulating the activity of genes related to fatty acid synthesis, inducing the regression of aortic lesions, and decreasing inflammation and oxidative damage in experimental animals [175]. Although previous evidence suggested no significant change in serum total cholesterol, LDL cholesterol was significantly lower in the berries-consumed than in the placebo-treated subjects [176]. To complement this, a meta-analysis and trial sequential analysis of randomized controlled human trials was evaluated [177]. The authors established the potential role of CRP in cardiovascular disease since CRP can bind to LDL cholesterol and is present in atherosclerotic plaques. In this line, berry consumption markedly diminished CRP and TNF-α levels, decreasing inflammation and preventing the development of cardiovascular disease.

In a recent study [178], the authors established that proanthocyanidins regulate blood pressure due to antioxidative scavenging of oxidized LDL and LDL cholesterol and the removal of carotid atherosclerosis plaque. Oxidative stress may be reduced by protecting the blood–brain barrier during arteriosclerosis by inhibiting oxidized LDL docking to its receptor LOX-1 to prevent cerebrovascular diseases. Proanthocyanidin extract (0.1–1%) incorporated into rabbit diets ameliorated cholesterol-induced aortic lesions and atherosclerosis [179] while also decreasing oxidized LDL activation and foam cells via the antioxidative mechanism.

The benefits and importance of proper daily fruit consumption for health are widely known. In this line, mulberry has been demonstrated to inhibit the oxidation of LDL and reduce the intracellular ROS generation of macrophages. Mulberry leaf extract and mulberry leaf polyphenolic extract exhibit strong antioxidant properties, effectively neutralizing free radicals and lipid peroxides. Both improved the expression of antioxidant enzymes (superoxide dismutase-1, catalase, and glutathione peroxidase), lowered the expression of scavenger receptors via downregulating the transcription factor PPAR-γ, inhibiting the oxidized LDL uptake, foam cell formation, and intracellular lipid accumulation [180].

On the other hand, polyphenols can be implicated in a bidirectional relationship with gut microbiota affecting each other. According to Filosa et al. [181], polyphenols undergo enzymatic transformation by the microbiota, leading to enhanced bioavailability and improved health. In turn, polyphenols also influence the composition of the microbiota, preventing the proliferation of pathogens. Specific polyphenols can inhibit/increase the development of particular bacteria resulting in modulation of gut microbial composition [182]. Polyphenols can enhance the abundance of beneficial bacteria, such as Bifidobacterium and Lactobacillus, which contribute to gut barrier protection, *Faecalibacterium prausnitzii,* which presents anti-inflammatory action by blocking NF-kB activation, and *Roseburia* sp., which are butyrate producers.

In this line, probiotics administered in appropriate doses offer positive effects for the host [183]. Some bifidobacteria and lactobacilli prevent the adhesion of pathogenic bacteria by secreting lectin-like bacteriocins. The barrier protective effect involves the release of metabolic or other molecules, which, in turn, regulates tight junction integrity [184]. According to Gou et al. [185], lactobacillus plantarum MB452 increases the gene and protein expression of zonula occludens-1, zonula occludens-2, occludin, and cingulin. It also regulates the expression of tight junctions’ protein-degrading genes, stabilizing tight junctions and improving intestinal barrier function. As well, *Bifidobacterium infantis* and *Lactobacillus acidophilus* normalize the expression of the tight junctions’ proteins, occludin and claudin1, in an in vitro Caco-2 intestinal epithelial cell model, preventing barrier damage due to IL-1 stimulation. In this line, a six-week wild blueberry powder drink intake can positively modulate intestinal microbiota composition by increasing bifidobacterium [186].

**Figure 2:**
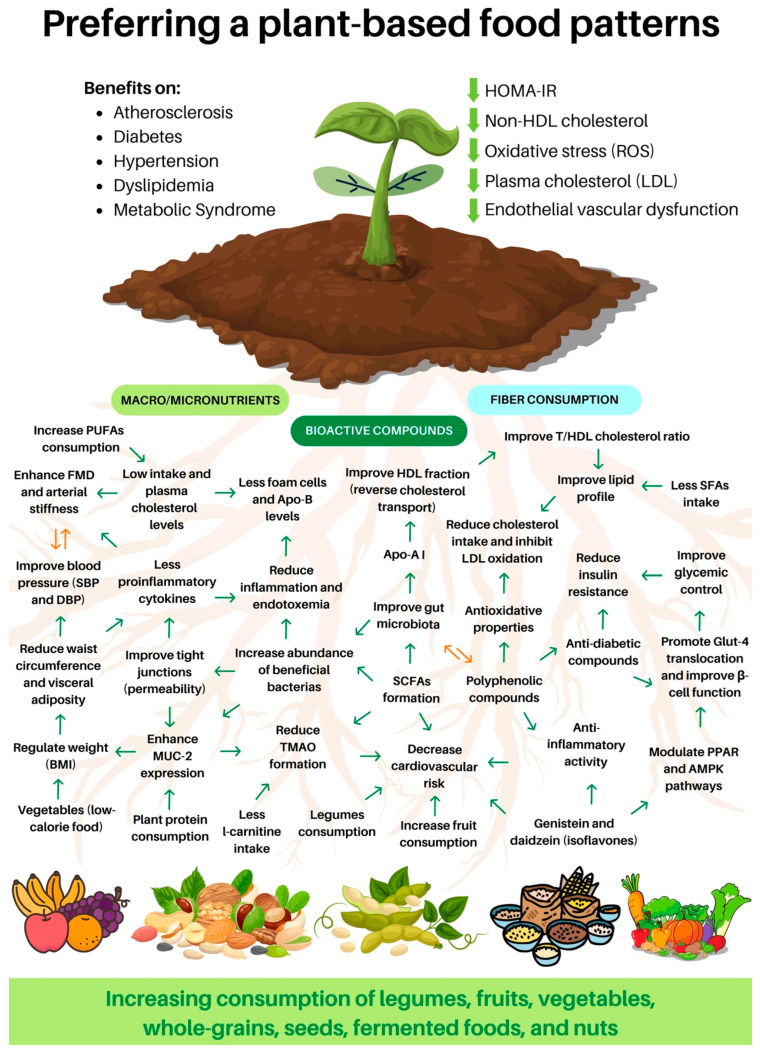
Benefits of consuming a plant-based diet. TMAO: trimethylamine N-oxide; FMD: flow-mediated dilatation; SFAs: saturated fatty acids; PUFAs: polyunsaturated fatty acids; HDL: high-density lipoprotein; LDL: low-density lipoprotein; T/HDL cholesterol: total cholesterol and HDL cholesterol ratio; Apo: apolipoprotein; SBP: systolic blood pressure; DBP: diastolic blood pressure; BMI: body mass index; Glut-4: glucose transporter; SCFAs: short-chain fatty acids; PPAR: peroxisome proliferator-activated receptors; AMPK: AMP-activated protein kinase; ROS: reactive oxygen species; HOMA-IR: Homeostasis model assessment of insulin resistance; MUC-2: mucin-2.

Another bioactive compound with multiple health benefits is present in coffee. Previous evidence has investigated the relationship between coffee consumption and oxylipin, a biomarker related to cardiovascular disease, inflammation, and lipid peroxidation produced during foam cell formation in atherogenesis. The authors found that, after coffee consumption, urinary oxylipin was reduced. The phenolic compounds in coffee were implied to have anti-inflammatory and antioxidant activities. It is interesting to highlight that the participants received two coffees with different amounts of chlorogenic acid in this study. Those with a higher chlorogenic acid intake demonstrated higher oxylipin reduction, suggesting the protection of coffee against cardiovascular disease progression and development [187]. Finally, Table 1 displays a summary of findings on this matter and Figure 2 provides a comprehensive overview addressing the overall advantages of adopting a plant-based diet.

## 3. A Plant-Based Diet and Chronic Non-Communicable Diseases

### 3.1. Diabetes

Diabetes is a prevalent common chronic condition affecting the human population. According to The World Health Organization (WHO), diabetes is projected to rank as the seventh leading cause of mortality globally by the year 2030 [188]. The macro and microvascular complications associated with diabetes are more common in older adults than middle-aged people. According to this, macrovascular complications can lead to an impaired platelet function and, thus, an increased risk for thrombus formation, atherosclerosis progression, and plaque rupture [189]. This has been supported by Huang et al. [190] who found that some microvascular complications, such as nephropathy and retinopathy, are a negative consequence but are usually not detected until late in the course of cardiovascular disease. In a 5-year follow-up observational study that included nearly 3000 participants adhering to a vegetarian diet while abstaining from smoking and alcohol consumption, a 35% lower risk of the incidence of diabetes was observed. The transition to a vegetarian diet also decreased the incidence of diabetes by 53% [191].

In this line, a PBD represents an appropriate option by which to enhance antioxidant consumption [192]. These outcomes have been supported by a recent meta-analysis [193]. The strength of this relationship was amplified when the definition of plant-based patterns encompassed fruits, vegetables, whole grains, legumes, and nuts. Similar conclusions have been drawn elsewhere [194].

The above-mentioned foods share a common feature: bioactive compounds. Among these, flavonoids are known as anti-diabetic bioactive compounds [188]. This property is related to the modulatory effects on blood sugar transporters by enhancing insulin secretion, reducing apoptosis, promoting the proliferation of pancreatic β-cells, reducing insulin resistance, inflammation, oxidative stress in muscle, and promoting Glut-4 translocation via PI3K/AKT and AMP-activated protein kinase (AMPK) pathways [195]. One of the most common flavonoids, quercetin, induces activities similar to those of metformin in muscle cells by activating AMPK pathways and thereby causing Glut-4 translocation [196]. However, quercetin also reduce proinflammatory cytokines, such as TNF-α, IL-6, and IL-1b, and modulates certain transcriptional factors like NRf2 and NF-kB [197,198]. The latter is essential, considering that accumulative evidence suggests that chronic activating proinflammatory pathways in target cells of insulin action may contribute to obesity, insulin resistance, and related metabolic disorders, including diabetes [199].

On the other hand, polyphenols are mostly found in tea, cocoa, and fruits, such as apples, berries, and citrus, among other plant-derived foods. Polyphenols act in an insulin-dependent manner by reducing β-cell apoptosis and oxidative stress, whilst stimulating β-cell proliferation, insulin signaling, and pancreatic insulin secretion [200]. They also act in an insulin-independent manner by inhibiting glucose absorption, digestive enzymes, and the formation of advanced glycation end-products, whilst regulating intestinal microbiota and modifying the inflammatory response. Moreover, dietary polyphenols ameliorate diabetic complications, such as vascular dysfunction and coronary diseases, among others [200].

A previous meta-analysis found that a 5% decrease in type 2 diabetes risk was obtained by a daily increase in anthocyanidin intake (7.5 mg) [201]. However, some studies present inconclusive results. One possible explanation of their beneficial effects lies in PPARγ activation. Some plant phytochemicals, such as quercetin, resveratrol, genistein, or curcumin affect inflammatory cascades by activating AMPK signaling via proteasomal activation and by inactivating crucial transcription factors, which may explain most of the properties attributed to PPARγ interaction. PPARγ represses inflammatory gene expression as inducible nitric oxide synthase suppresses transcriptional factors AP-1 and NF-κB, modulates mitogen-activated protein kinase (MAPK) activity, and influences glucose uptake [202].

Genistein, daidzein, and glycitein are the most active isoflavones and are mainly found in soybeans. In fact, these bioactive compounds have been proven to reduce cancer and decrease the risk of some chronic diseases like type 2 diabetes. Evidence on isoflavones reducing type 2 diabetes risk is mainly derived from animal and cell culture studies [203,204]. Extrapolating these findings to humans should be done cautiously due to the inherent limitations and differences of the biological context. Nonetheless, isoflavones have also shown a positive effect in human trials. For instance, two months of genistein consumption (50 mg/d) reduced insulin resistance in obese individuals, accompanied by a favorable modulation of the gut microbiota composition. Furthermore, subjects showed reduced metabolic endotoxemia and increased AMPK phosphorylation and genes expression involved in fatty acid oxidation in skeletal muscle [205].

Additionally, a one-year intervention with flavan-3-ols and isoflavones (850 mg/d and 100 mg/d, respectively) markedly reduced estimated peripheral insulin resistance (HOMA-IR) and enhanced insulin sensitivity due to a notable decrease in insulin levels in post-menopausal women with type 2 diabetes [206]. Moreover, genistein improved insulin sensitivity, serum triglyceride concentrations, and delayed the onset of type 2 diabetes [207]. Previous evidence proposes that genistein may help delay the onset of type 2 diabetes and improve different associated symptoms [207].

Finally, the whole-food plant-based diet has repeatedly shown benefits in this context [193]. Three prospective cohort studies found an inverse association between a healthy plant-based diet and type 2 diabetes, with 16,162 incident cases observed over 4,102,360 person-years of follow-up. These positive associations are related to antioxidants, fiber, unsaturated fatty acids, magnesium, and low saturated fat content. This outcome remains unchanged after the authors adjusted for body mass index [208]. In another large sample, maintaining an overall PBD is linked with reduced longitudinal insulin resistance, prediabetes risk, and type 2 diabetes. Further, the authors also concluded that the protective role of this diet is beyond strict vegetarian or vegan diets and includes a high plant-based diet and fewer animal-option foods [209].

Other authors concluded that a PBD accompanied by educational intervention could significantly improve HbA1c levels, weight, and, therefore, diabetes management [210]. Jardine et al. [3] highlight that insulin resistance and the succeeding dysfunction in β-cell serve as the key characteristics of the pathophysiology underlying type 2 diabetes. Along with this, long-term intervention and even a single high-fat meal can cause postprandial elevations in plasma glucose that can remain high for an extended period. This result is similar to what was found by Parry et al. [211]. The authors specify that consuming a saturated fat diet had a strong effect, improving intrahepatic triacylglycerol and exaggerating postprandial glycemia. Thus, a PBD has the potential to reverse β-cell dysfunction and peripheral insulin resistance in patients with type 2 diabetes [212], partly by improving glycemic control, reducing lipid accumulation in muscle and liver, and/or improving insulin sensitivity. This is important since a multi-adjusted analysis revealed that baseline non-alcoholic fatty liver was associated with a 2.95 times higher risk of type 2 diabetes within 10 years. The evidence has shown that a healthy plant-based diet has an inverse association with non-alcoholic fatty liver. Conversely, an unhealthy plant-based diet, distinguished by low amounts of fiber, vitamins, or minerals, and more refined sugar or sodium consumption due to the increase in ultra-processed foods, showed the opposite results [213].

### 3.2. Hypertension

Hypertension may be considered a chronic disease and is a risk factor for other diseases. Its incidence depends on modifiable risk factors (e.g., smoking, diet, drinking, or sedentarism/physical inactivity) and non-modifiable risk factors (e.g., genetic predisposition). As such, a diet or following a healthy foods pattern can be crucial for prevention and treatment. A healthy plant-based diet has shown positive results in both hypertensive and non-hypertensive people. In a 3-year prospective study with 1546 non-hypertensive individuals spanning the age range of 20 to 70, higher phytochemicals-rich foods consumption was linked with a lower risk of developing hypertension [214]. In a large study with 13,771 participants, the authors demonstrated that only in male individuals was an increase in antioxidants, vitamins, and phytochemicals correlated significantly with a reduction in CRP, systolic, and diastolic blood pressure (SBP/DBP, respectively) [215].

Previous studies showed that vegetarians, especially vegans, have lower SBP and DBP, and less hypertension than omnivores [216,217]. In a 4-week study, participants changed their diets to a PBD. The authors observed changes in nutrient intake, reducing saturated fat, cholesterol, protein, and some vitamins and minerals such as sodium, vitamin D, or vitamin B12. However, after four weeks, vitamin C, folate, dietary fiber, magnesium, vitamin A, and potassium significantly increased. Different measured biomarkers changed significantly, such as total cholesterol, LDL cholesterol, HDL cholesterol, triglycerides, insulin, HbA1c, and hs-CRP. However, glucose levels and total cholesterol/HDL ratio remained unchanged. The main findings highlight that SBP and DBP clinically and significantly changed after four weeks in a PBD. Interestingly, there were notable and marked declines in both blood pressure medication usage and serum lipids [218].

Berries can positively affect vascular function and reduce hypertension via their phytochemical anthocyanins. Previous studies have reported that a higher intake of anthocyanins and flavones is inversely associated with lower arterial stiffness in women through the significant reduction in central SBP, suggesting that incorporating one to two portions of berries daily (44 mg anthocyanins) could be relevant to reducing cardiovascular disease risk [219]. Fairlie-Jones et al. [220] stated that anthocyanins consumption from foods or extracts significantly enhanced vascular health. FMD, as an indicator of vascular function, is considered the gold-standard non-invasive vascular reactivity measure and has improved after acute or chronic anthocyanidins consumption.

Supporting this, other authors have highlighted that anthocyanin is an inexpensive, accessible, and effective approach to controlling atherosclerosis, cardiovascular risk, and cardiovascular aging. They can exert their effects mainly by improving endothelial function, managing oxidative stress, and inhibiting certain enzymes such as cyclooxygenase-1 and cyclooxygenase-2, which exert antihypertensive, antiatherogenic, antithrombotic, antiglycation, and anti-inflammatory activities, and ameliorate dyslipidemia and arterial stiffness [221]. Furthermore, a recent study [178] claimed that the main mechanism is an increase in endothelial-derived nitric oxide, which enhances endothelium-dependent vasorelaxation and prevents calcium-induced vascular smooth muscle contraction induced by endothelial nitric oxide synthase.

Conversely, high dietary sodium consumption is one of the significant harmful agents that impairs blood pressure homeostasis. According to the WHO, an intake of 5 g or more of sodium per day is considered excessive. This has been strongly connected to elevated blood pressure and the start of hypertension and its related cardiovascular complications [222].

The International Society of Hypertension Global Hypertension Practice Guidelines recommend reducing dietary salt intake and increasing the availability of fresh fruits and vegetables. The support for this lies in the physiological function of potassium in sodium homeostasis [223,224]. However, not all evidence supports this association [225]. An earlier study explained the theoretical mechanism referring to the capacity of potassium to modify central or peripheral neural mechanisms that regulate blood pressure. Additionally, high-potassium diets can lower blood pressure by relaxing the vascular smooth muscle and directly decreasing peripheral vascular resistance [226].

Furthermore, the “Renal Potassium Switch” (RPS) can be activated or inhibited depending on dietary potassium consumption. When potassium consumption is low, RPS activates the NaCl cotransporter (NCC) in the distal convoluted tubule. Interestingly, increased potassium intake decreases blood pressure by ~10 mmHg, which coincides with NCC inactivation. A high salt intake in animals significantly elevated blood pressure but not when the RPS was inactive [227,228]. Along with this, another study supported the positive function of potassium in regulating blood pressure and/or hypertension [229].

It was explained previously that small changes in serum potassium can cause endothelium-dependent vasodilation by hyperpolarizing the endothelial and vascular smooth muscle cells. A high-potassium diet may also enhance vascular integrity on increased tension due to hypertension. A high-potassium diet substantially reduced wall thickening in the very large or small arteries of spontaneous hypertension rats (SHR). In a previous analysis, a four-week increase in potassium consumption potentiates AngII-stimulated aldosterone secretion without affecting systemic cardiovascular hemodynamics in healthy normotensive men. This indicates that the antihypertensive effect of potassium is mediated by its diuretic effect [230]. In addition to maintaining normal plasma potassium levels, these natriuretic effects contribute to the blood pressure-lowering effect of high potassium intake. The result of a potassium-deficient diet at the expense of increased sodium retention has been linked to the pathogenesis of salt-sensitive hypertension [231].

A PBD may reduce blood pressure, body mass index, and lower sodium levels whilst increasing potassium content in foods. Also, its function has been analyzed in terms of improving NO bioavailability and the beneficial effect on the microbiome [5]. A recent study highlights the relevance of opting for a healthy plant-based diet in preventing hypertension. The authors found that 2244 individuals, based on a community cohort of 5636 men and women between 40–69 years, developed hypertension in a 14-year follow-up period. However, according to a healthy plant-based diet, the highest versus lowest quintiles exhibited significant differences. Those in the highest quintile of a healthy plant-based diet had a 35% lower incidence of hypertension, while an unhealthy plant-based diet showed a 44% greater hypertension incidence [232].

### 3.3. Dyslipidemia

Koh et al. [233] identified dyslipidemia as a prominent risk factor for ASCVD, a condition marked by the imbalance of atherogenic and protective lipids, such as triglycerides, LDL cholesterol, and HDL cholesterol. Statins are standard treatment, as they inhibit the critical step in cholesterol synthesis: the conversion of 3-hydroxy-3-methylglutaryl coenzyme A (HMGC) to mevalonate by HMGC reductase. This gives statins a potent lipid-lowering effect that reduces cardiovascular risk and decreases mortality (reducing LDL cholesterol by ≥50%). However, statins have many common side effects ranging from musculoskeletal symptoms, increased risk of diabetes, and higher rates of hemorrhagic stroke [234].

Considering that mentioned above, a PBD has shown multiple beneficial effects on dyslipidemia without adverse side effects. In a recent pilot, dietitian-led, vegan 12-week program, the results showed a significant reduction in LDL cholesterol and LDL particles, with a decreasing trend in very low lipoprotein density and chylomicron particles. These beneficial changes have been attributed to the observed decrease in inflammation, as measured by GlycA [235]. GlycA is considered a novel marker related to systemic and subclinical vascular inflammation [236], a significant consequence of dyslipidemia that affects others’ pathogenesis. In a study of 38 Romanian subjects who adopted a PBD for at least one year, the authors observed that 75% of subjects with elevated TG succeeded in normalizing them, as well as individuals with high LDL cholesterol levels, where 72.7% from the borderline elevated level became optimal. The total cholesterol/HDL ratio shifted from elevated to optimum levels in 78.6% of the cases [237].

One of the plant-based source components that plays an essential role in health is fiber. Different studies have evaluated dietary fiber’s benefits for reducing LDL cholesterol. In 1999, with 67 controlled clinical trials, it was reported that other soluble fibers could reduce total and LDL cholesterol to a similar extent. For instance, the consumption of 3 g of soluble oat fiber reduces LDL cholesterol by <0.13 mmol/L [238].

A recent meta-analysis found that foods high in unsaturated and low in saturated and trans fatty acids with added plant sterols/stanols and high in soluble fiber at least moderately reduce LDL cholesterol [239]. This has also been published in a previous study concluding that a low glycemic index and at least 23 g of fiber a day can help to improve dyslipidemia in subjects with type 2 diabetes. A particular finding in this study was that the changes were not dependent on altering energy intake or body composition. This is important because most beneficial effects could eventually be overshadowed or mixed with improving body composition, particularly the decrease in fat mass or improvement in the percentage of total body fat [240].

The mechanism by which fiber improves cardiovascular health is not fully understood. It has been proposed that fiber could modify cholesterol metabolism by directly interacting with pancreatic lipase, binding to bile acids, increasing intraluminal viscosity, intestinal microbiota secreting fermentation products that modulate hepatic fatty acid synthesis, changes in intestinal motility, and increasing satiety that results in to lower overall energy intake [238,241]. Soluble fiber directly affects serum cholesterol and LDL cholesterol levels by binding bile acids in the small intestine and increasing their excretion in the feces [242]. This trapping of cholesterol and bile acids in the small intestine reduces absorption/reabsorption [243]. SCFA complements this effect.

Soluble fiber is resistant to hydrolysis in the small intestine but is fermented by gut microbiota in the large intestine. In this context, a previous study observed that SCFA reduces cholesterol levels by negating the counteractive induction of hepatic cholesterol synthesis caused by increased bile acid excretion [244]. Also, it appears that SCFA plays a role in the production of Apo-A I, which could consequently improve the functionality of the serum HDL fraction [245]. This is relevant as HDL performs the opposite action to LDL (reverse cholesterol transport). The absorption of SCFAs such as propionic acid has been shown to decrease cholesterol synthesis in the liver, thus reducing plasma cholesterol and increasing sodium and water absorption into the colonic mucosal cells.

Further, dietary saponins directly inhibit cholesterol absorption in the small intestine and indirectly inhibit the reabsorption of bile acids to lower plasma cholesterol. Additionally, phytochemicals have been shown to decrease LDL levels, which signal cholesterol build-up, thus indicating that phytochemicals can also be used to reduce blood cholesterol levels [246]. Therefore, if cholesterol absorption from the diet is reduced, physiological adaptations must be generated to maintain levels within normal ranges. Lütjohann et al. [247] observed that lacto-vegetarians absorbed 44% less dietary cholesterol but synthesized 22% more cholesterol, while vegans absorbed 90% less dietary cholesterol, synthesized 35% more cholesterol, and had a similar plasma total cholesterol but a 13% lower plasma LDL cholesterol than omnivores. The authors concluded that the reduction of LDL cholesterol was significant only in vegans.

On the other hand, not all studies found a PBD to be beneficial for hypertriglyceridemia [9]. However, the outcomes were positive when specific foods such as walnuts were tested. Indeed, walnut consumption has shown notable improvements in triglyceride levels, particularly among overweight/obese individuals, with men experiencing more significant results than women. Nonetheless, a subgroup analysis reflects much-lowering effects, comparing individuals with comorbidities versus healthy subjects [248]. Thus, the different or negative impacts could be related to the “type of diet”.

As we mentioned at the beginning, according to the literature, two types of PBD exist: healthy and unhealthy. In this line, a plant-based diet index (PDI), which separates healthy plant-based diet options (i.e., fruits, whole grains, vegetables, legumes, nuts, coffee, and tea) from less healthy plant-based diet options (i.e., refined grains, potatoes, sugar-sweetened beverages, sweets and desserts, salty foods), was evaluated. The authors found that those in the highest quintile of PDI and consuming a healthy plant-based diet consumed more carbohydrates (including fiber), vitamins, and minerals but less cholesterol, protein, and fat. In this study’s follow-up of 29,313 person-years, the incidence of dyslipidemia was significantly lower in healthy plant-based diets than in unhealthy plant-based diets, comparing the highest and lowest quintiles. In fact, one standard deviation of PDI and healthy PDI was associated with a 9% and 16% lower risk of incident dyslipidemia, respectively, and one standard deviation of unhealthy PDI was associated with a 16% higher risk of dyslipidemia after adjustment for confounders effects. While PDI and a healthy plant-based diet were inversely connected with hypertriglyceridemia, an unhealthy plant-based diet was associated with all lipid disorders. Interestingly, this strong association remained after adjustment for anti-dyslipidemia medication [249].

**Figure 3:**
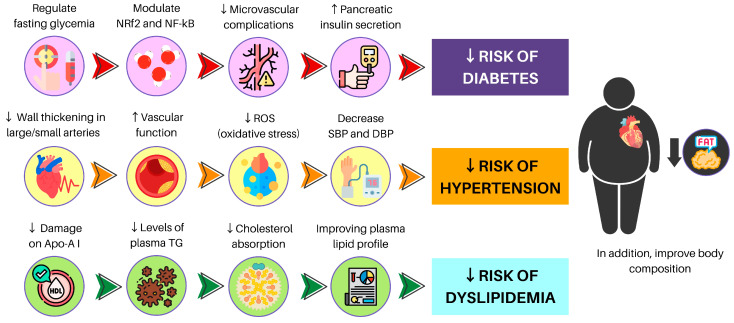
A brief description of PBD benefits for NCCD and MetS. NRf2: nuclear factor erythroid 2-related factor 2; NF-kB: nuclear factor-κB; ROS: reactive oxygen species; SBP: systolic blood pressure; DBP: diastolic blood pressure; Apo-A I: apolipoprotein A I; TG: triglycerides.

Additionally, previous evidence has shown that quinoa could decrease weight gain, improve lipid profile, and improve capacity to respond to oxidative stress, mainly due to saponins content [250]. Another study also showed decreased LDL cholesterol and TG after 30 days of quinoa bar consumption [251]. This has been supported previously [252]. Here, the authors found a 36% reduction in TG levels after 12 weeks of 50 g of quinoa consumption. The mechanism by which quinoa exhibits these benefits is not fully comprehended. However, their effects are related to fiber content, reduction in dietary fat absorption due to increased lipid content in feces, and/or bile acid activity. One crucial detail is that these changes in triglyceride reduction levels were comparable to the reduction evidenced in pharmacologic therapy that used 40% nicotinic acid, 35% fibrates, and 20% statins. Finally, Table 2 displays a summary of findings on NCCD and MetS, Figure 2 provides a comprehensive overview addressing the overall advantages of adopting a plant-based diet, and Figure 3 provides a synthesis addressing the general effects of adopting a plant-based food pattern against specific chronic non-communicable diseases.

## 4. A Plant-Based Diet and Metabolic Syndrome

### 4.1. Insulin Resistance (Fasting Blood Sugar)

The relationship between high fasting blood sugar (hyperglycemia) and insulin resistance (IR) is consistent. IR is a pathological condition in which cells fail to respond normally to insulin. Consequently, this leads to elevated blood glucose levels, often accompanied by hyperinsulinemia and hyperglycemia. In this condition, pancreatic β-cells secrete excessive insulin to maintain normal blood sugar levels.

The principal function of insulin in skeletal muscle is to stimulate glucose uptake by inducing the translocation of the specific glucose transporter GLUT4 from the cytoplasm to the plasma membrane. Upon binding to GLUT4, insulin initiates a signaling cascade to phosphorylate and activates the Insulin Receptor Substrate (IRS), leading to the activation of various protein kinases (PI3 kinase, Pdk1, and Akt). Activated Akt moves to the plasma membrane and phosphorylates Akt Substrate of 160 kDa (AS160). AS160 initiates GLUT4 translocation, allowing for glucose influx into skeletal muscle. This glucose is then undergoing glycolysis and thus blood glucose levels are lowered.

A previous study has demonstrated that both acute blueberry consumption and short-term blueberry supplementation have beneficial effects on glucose regulation and insulin balance in sedentary individuals, presumably mediated through gastrointestinal enzyme inhibition and incretins secretion [271]. Prioritizing plant sources to the detriment of traditional animal alternatives results in lower IR and a lower risk of prediabetes and type 2 diabetes [272]. Similarly, Kahleova et al. [212] found in a 16-week randomized clinical intervention that β-cell function and fasting insulin resistance were improved by a qualitative change in macronutrient composition with no limit on energy intake in overweight individuals with no history of diabetes. HOMA-IR is considered an effective indicator of IR since it provides an estimate of fasting glucose and insulin serum concentrations [273].

Further, previous studies have extensively demonstrated that AMPK stimulation can improve health in different contexts. The AMPK signaling pathway provides an alternative to the insulin-dependent glucose uptake pathway in muscle by activating phosphatidyl-inositol-3 kinase (PI3-K) and PKB/Akt [253]. Data support that AMPK inhibits Rab-GTPase activating proteins AS160 (TBC1D4) and TBC1D1, which triggers Glut-4 trafficking to the plasma membrane [274]. Firstly, related to the stimulation of Glut-4 translocation and AMPK activation, a previous study demonstrated that anthocyanin-rich extract from black rice promotes glucose uptake by increasing Glut-4 expression in the plasma membrane in C2C12 myotubes via activation of the PI3K/Akt and AMPK/p38 MAPK pathways [275]. Furthermore, resveratrol, a naturally occurring phytochemical, increases glucose uptake in insulin-resistant 3T3-L1 adipocytes by increasing pAkt phosphorylation and downstream AMPK activation. In another study, berberine, a natural plant-derived compound, can indirectly activate AMPK. Furthermore, a berberine derivative demonstrated enhanced insulin sensitivity and reduced adiposity in vivo in high-fat diet rats [254].

On the other hand, in recent years, PPAR has gained significant interest due to its potential role as an essential regulator of glucose metabolism and insulin sensitivity. PPAR-α activation stimulates pancreatic islet β-cells, potentiating glucose-stimulated insulin secretion. On the contrary, PPAR-α deficiency in a mouse model of obesity-related insulin resistance leads to reduced insulin secretion by pancreatic β-cells in response to glucose [255]. This improvement in insulin sensitivity has been theorized in the modification of signaling due to a decrease in ectopic lipids in non-adipose tissues and a decrease in circulating fatty acids and triglycerides, seen in animal models [256]. Furthermore, PPARγ activation in type 2 diabetic patients results in a marked improvement in insulin and glucose parameters by modifying whole-body insulin sensitivity.

Given this, diet-induced PPAR downregulation can be viewed as a negative effect. For instance, a high-fat diet can negatively affect the activity or expression of PPAR, favoring several complications, such as IR. However, some bioactive plant components, like the polyphenols present in coffee, rice, berries, and others, can modulate this pathway, increasing PPARα and γ mRNA [257]. Polyphenols have a positive effect on insulin sensitivity and improve IR by way of several mechanisms, including lowering postprandial glucose, modulating glucose transport, affecting insulin signaling pathways, and protecting against damage to insulin-secreting pancreatic β-cells [276]. In fact, synergistic metabolic action between exercise and polyphenols consumption from grapes counteracts anthropometric and metabolic impairments. It increases insulin sensitivity, probably via lipid oxidation enhancement and glycogen utilization reduction. Although this study was performed in animals, the amount of polyphenols consumed by the specific grape is equivalent to nutritional amounts ingested daily by humans [258].

Moreover, in a study of IR nondiabetic adults, the 6-week consumption of strawberry and cranberry polyphenols showed that the consumption of 333 mg polyphenols might improve insulin sensitivity and prevent an increase in compensatory insulin secretion without affecting plasma lipids, CRP, proinflammatory cytokines, or antioxidant capacity [270]. This result is in line with a previous study that evaluated daily dietary bioactive supplementation in freeze-dried whole blueberry powder. The authors found an improvement in insulin sensitivity in obese, nondiabetic, and insulin-resistant participants after six weeks, independent of any changes in inflammatory biomarkers or adiposity [277]. Different other studies have found similar results [278,279].

Inflammatory signaling pathways are associated with the activation of TLRs. TLRs are transmembrane proteins that regulate the innate immune response in various pathophysiological states like sepsis and cardiovascular disease [280]. TLR signaling triggers transforming growth factor β activating kinase 1 activation and, subsequently, also MAPK and NF-ĸB [281]. NF-ĸB regulates the transcription of inflammatory cytokines such as TNF, IL-6, and IL-1 [282]. TLR2 and TLR4 are involved in inflammation and insulin resistance [283] in human skeletal muscle cells [284]. Interestingly, in mice, the absence of TLR2 and TLR4 from the plasma membrane protects against obesity and IR [285], which generated a lot of interest in the TLRs as possible therapeutic targets in the fight against obesity and IR. A recent review identified some plant extracts as potential modulators of TLRs controlling inflammation and the immune response [259]. Counteracting the negative effects of chronic low-grade inflammation may result in beneficial effects in different pathological states such as insulin resistance.

### 4.2. Visceral Obesity and Waist Circumference

A recent study reported that greater adherence to a healthy plant-based pattern, but not an unhealthy one, was linked with lower visceral adipose tissue, accounting for several potential confounding variables [260,286]. This outcome aligns with a Dutch study comparing sweet snacks against fruit and vegetable consumption. While the first was associated with hepatic triglyceride content, consuming fruits and vegetables was negatively related to visceral fat and liver fat content (triglycerides) [261]. The theoretical mechanism of these positive results is partly in energy consumption, considering that a PBD is high in fiber [287] and higher in antioxidants. In the case of fiber, it can help increase satiety with little or no calorie intake. 

On the other hand, antioxidants can reduce inflammation related to visceral adiposity. A healthy and unhealthy plant-based diet have marked differences in the amounts of sugar, glycemic index, and energy intake with the theoretical capacity to increase weight and, consequently, waist circumference.

A recent study [263] performed on older Australian women showed that body weight, body mass index, and waist circumference were significantly lower in pesco-vegetarian and vegetarians than meat-eaters. Moreover, among meat-eaters, subjects that consume meat regularly or several times a week, compared with those consuming meat two or fewer times a week, present higher body weight, body mass index, and waist circumference. To be detailed, in a dose-dependent meat intake, for every increase in the category of weekly meat intake, an associated 2.6 kg increase in body weight, a 0.9 kg/m^2^ increase in body mass index, and a 2–3 cm increase in waist circumference were reported. Although this evidence does not qualify as a PBD, it is essential to highlight that meat consumption frequency was the primary factor involved in the main results. Another study that included 9633 participants showed that greater adherence to a PBD was associated with lower body mass index, waist circumference, fat mass index, and body fat percentage across a median follow-up period of 7.1 years [262]. This result has been previously supported by other authors [288].

### 4.3. High-Blood Pressure

High blood pressure is universally known as a risk factor for several chronic diseases, such as hypertension. In a recent meta-analysis [6], the authors concluded that a PBD reduced SBP and DBP. The authors established that a high fruit and vegetable diet was associated with a mean reduction in SBP. Although some studies did not observe a consistent association between fruit and vegetable consumption and improved blood pressure in older age [264], the central body of evidence has found positive results. For instance, healthy PDI is associated with lower blood pressure, while unhealthy PDI is adversely related to blood pressure. A PBD rich in vegetables and whole grains and limited in refined grains, sugar-sweetened beverages, and total meat may contribute to these associations [289]. In a study by Stefler et al. [264], the preference for vegetable consumption involves preserved or cooked vegetables instead of having them raw, and including salt during both types of procedures might counteract the positive benefits of these foods. Additionally, the authors mentioned some study limitations that could explain the lack of clear association. Nevertheless, other studies have found the benefits of fruit and/or vegetable intake for blood pressure.

In this line, it has been hypothesized that fiber, different nutrients, bioactive components, and plant-sourced foods, such as fruits and/or vegetables, can exert their benefits on blood pressure due to the improvement in baroreflex sensitivity [290], endothelial-dependent vasodilatation related to high-flavonoid intake [291], and the inhibition of inflammation and oxidative stress response [265,266]. In a study on cranberry juice and vascular function, patients with coronary artery disease registered a reduction in carotid-femoral pulse wave velocity, a clinically relevant measure of arterial stiffness [292].

### 4.4. Hypertriglyceridemia

A PBD has widely demonstrated several benefits related to MetS. For instance, in a recent South Korean prospective cohort study [293] comparing the highest with the lowest quintiles of an unhealthy plant-based diet linked to greater intake versus lower intake, in a follow-up of 8 years, the highest quintile had a 50% higher risk of developing MetS. After adjusting for body mass index, those in the highest quintile of an unhealthy plant-based diet had a 24% to 46% higher risk of four out of five individual components of MetS, including hypertriglyceridemia. These results have been recently supported, detailing that greater adherence to PDI or healthy PDI was associated with a lower risk of incident dyslipidemia. In contrast, greater adherence to unhealthy PDI was associated with a higher risk.

Moreover, the authors detailed that PDI was inversely associated with low HDL cholesterol among women, while among men a greater adherence to PDI was inversely associated with hypertriglyceridemia [249]. Based on Korean food patterns, the authors reported that the lipid composition from a PBD leads to less absorption and conversion to blood cholesterol and reduced triglyceride concentration. This is because plant foods are rich in compounds for preventing dyslipidemia, such as dietary fiber, phytosterols, antioxidants, and polyphenols.

On the other hand, although some studies did not refer to a PBD, the reduction in animal-based food has also shown positive effects. Teixeira et al. [294], in a comparison between vegetarians and omnivores, found that different indicators such as blood pressure, fasting plasma glucose, total cholesterol, LDL cholesterol, and triglycerides were lower among vegetarians. In fact, the authors complemented the results, highlighting that an unbalanced omnivorous diet with excess animal protein and fat may be implicated, to a great extent, in the development of non-communicable diseases and conditions, especially cardiovascular risk.

The consumption of dietary fiber of those who opt for a PBD and those who follow a traditional Western diet differs significantly. In this context, dietary fiber has been evaluated in the reduction of postprandial triglycerides response [295]. The beneficial mechanism of this property is strongly attributed to its viscosity, which effectively slows down gastric emptying and disrupts the emulsification of fats and the formation of micelles in the gastrointestinal tract. This is achieved by reducing the availability of circulating bile acids. Also, soluble fiber can be fermented by the gut microbiota to release metabolites such as SCFA, which upregulate genes PPARα and PPAR-γ coactivator-1α (PGC-1α) involved in lipid metabolism and the regulation of postprandial triglycerides response.

### 4.5. Dyslipidemia (Low HDL Cholesterol)

Generally, HDL cholesterol is considered a healthy biomarker due to its ability to reverse cholesterol transport and reduce cardiovascular risk. This cardioprotective function is supported by theoretical mechanisms, such as the regulation of cholesterol efflux, where apolipoprotein A-I (Apo-A I), the main component of HDL, is mainly responsible for reverse cholesterol transport through the macrophage ATP-binding cassette transporter ABCA. Another mechanism lies in the modulation of inflammation, antioxidant, and vasoprotective effects [296]. However, according to Kosmas et al. [297], HDL functionality is more critical in atheroprotection than circulating HDL cholesterol levels. In fact, plasma HDL constitutes a heterogeneous group of particles with diverse structures and biological activity and, under certain conditions, may become proinflammatory. Further, Apo-A I can be damaged by oxidative mechanisms, which render the protein less able to promote cholesterol efflux. Navab et al. [296] complement this information by pointing out that a modification of the protein components of HDL can convert it from an anti-inflammatory to a proinflammatory particle.

Very high HDL cholesterol could be more harmful than harmless; several previous investigations have reported that the elevation of HDL cholesterol could result in a cardiovascular risk factor [298,299]. This relationship between high HDL cholesterol levels and mortality risk has been observed even at >80 mg/100 mL, although the authors reported that it was related to men, not women [300]. Both extremely high and low HDL cholesterol are associated with an increased mortality risk [301].

These paradoxical results’ mechanisms and theoretical explanations can be attributed to two main factors. Firstly, some genetic disorders raise HDL cholesterol, such as primary familial or secondary hyperalphalipoproteinemia, mainly resulting in the overproduction or variants of apolipoprotein C-III (Apo-C III) due to a mutation in Apo-A I. These complications promote HDL cholesterol dysfunction, stimulate smooth muscle cell proliferation, facilitate the interaction between monocytes and endothelial cells, and alter platelet activity, all triggering atherosclerosis [298,299,301]. Secondly, a previous study [302] has shown that a moderate to high HDL concentration impairs human endothelial progenitor cells and related angiogenesis by activating rho-associated kinase pathways in healthy subjects. The authors found that, although oxidized LDL reduces the cell viability of late-growing EPCs derived from healthy human peripheral blood in a dose-dependent relationship, HDL alone might not be enough to counteract this negative effect and might even be paradoxically impaired at high concentrations (400–800 μg/mL, equivalent to 40–80 mg/dL in humans).

Some evidence has indicated that vegans, vegetarians, or PBD reduce total, LDL, and HDL cholesterol compared to omnivores in observational and clinical studies [9]. Along with this, some authors have found that a short-term, very low carbohydrate diet has been associated with an increase in HDL cholesterol in normal-weight normolipidemic women [303]. However, a low carbohydrate plant-based diet (eco-atkins) was evaluated in hyperlipidemic subjects. The result demonstrated a greater reduction in Apo-B, Apo-A I, LDL, and HDL cholesterol compared to a high carbohydrate diet, resulting in better context in which to improve heart disease risk factors [304]. This discrepancy of findings might generate a confounding analysis in evaluating a PBD, cardiovascular risk, and HDL cholesterol.

Regarding these findings, some authors have evaluated the total cholesterol/HDL cholesterol ratio (T/H-r) as a predictor of cardiovascular disease. Previous studies established that this ratio has a better predictive value than the isolated parameters in vascular risk. In fact, when there is no reliable calculation of LDL cholesterol, it is preferable to use the T/H-r [305]. An earlier study has supported this and indicated that T/H-r is a superior measure of risk for coronary heart disease compared with either total cholesterol or LDL cholesterol levels [306]. A recent large study [267] has sustained this finding. Although non-HDL cholesterol is linearly related to ischemic heart disease and may be easier to calculate, T/H-r represents a higher predictability as a clinical predictor than non-HDL cholesterol. In addition, Quispe et al. [307] reported that the potential clinical utility of T/H-r is most apparent among individuals in whom TC/HDL-C levels are inconsistent with their other lipid parameters (i.e., LDL cholesterol and non-HDL cholesterol). Discordance was more pronounced in those with high triglycerides and low HDL cholesterol levels, characteristic of patients with insulin resistance, diabetes, and MetS, who also have a higher prevalence of LDL and Apo-B particles that are cholesterol depleted.

Considering the information above, the lower T/H-r, the better. Bleda et al. [308] found that improvement of nitrite levels is associated with decreased T/H-r values in peripheral artery disease patients, resulting in endothelial dysfunction recovery. In this line, a previous study demonstrated that a low fat, plant-based lifestyle intervention reduced HDL levels but this was not as great as the decrease in LDL and TC, resulting in improvements in the T/H-r of −3.2% and the LDL/HDL ratio of −5.3% [309]. According to the authors, even when HDL levels decreased, other indicators of cardiovascular risk improved, raising the question of whether HDL levels are a suitable predictor of cardiovascular risk in this population.

In subjects who do not consume a typical Western diet or have a diagnosis of MetS, it may not be appropriate in clinical practice or research to apply lifestyle interventions that promote a plant-based eating pattern. It is relevant to mention that, although 323 participants classified as having MetS at program entry no longer had this status after 30 days, 112 participants acquired the MetS classification because of reduced HDL levels. Regarding HDL levels, the mean value changed by −8.7% from 54.84 (baseline) to 50.07 (post-intervention), considering the average value within the normal ranges. Supporting this, a previous meta-analysis suggested that the T/H-r was more predictive than HDL or non-HDL cholesterol sub-fractions and two times more predictive than total cholesterol [310].

Taking this into account, HDL has components with anti-inflammatory functions, such as Apo-A I and paraoxonase 1. At the same time, the proinflammatory action lies mainly in Apo-A II and Apo-C III. Regarding the latter, four prospective cohort studies indicated that Apo-C III might mark a subfraction of HDL associated with a higher risk of coronary heart disease [311]. This finding raises the question of whether the reduction in HDL may decrease in part the proinflammatory component of this particle.

The presence or absence of Apo-C III in HDL could be responsible for opposite outcomes related to cardiovascular risk. For instance, HDL that lacks Apo-C III inhibits the monocyte-endothelial cell interaction, leading to a lowered inflammatory response. In contrast, HDL with Apo-C III did not diminish this interaction [268]. Previous studies have provided supporting evidence that certain drugs aimed at improving health can increase HDL cholesterol levels but do not effectively reduce coronary atherosclerosis [269]. In this study, using the CEPT (cholesteryl ester transfer protein) inhibitor after one-year of treatment increased HDL cholesterol by approximately 60%, while LDL cholesterol decreased by about 20%. However, there was no significant reduction in the progression of coronary atherosclerosis according to the percent atheroma volume, the primary efficacy measure. Another study has found similar results with a different drug [312]. Given that, for people who follow a healthy plant-based diet, reducing HDL cholesterol could not be as harmful as those who suffer from a chronic disease. The decrease in HDL cholesterol is likely less of a concern and the need to increase these levels is less critical.

Nonetheless, some plant foods have shown a positive relationship with improving HDL cholesterol levels. In a randomized, single-blinded, controlled clinical trial [313], tomato consumption showed an independent positive association with HDL cholesterol. In an isocaloric diet, subjects were randomized to receive 300 g of cucumber (control group) or two uncooked Roma tomatoes daily for four weeks. The results indicated that subjects with initial low HDL cholesterol who ate tomato changed their levels from 36.5 ± 7.5 mg/dL to 41.6 ± 6.9 mg/dL. Another study evaluating a specific plant food also found a positive association [314]. The authors found that 10 g/day consumed before breakfast can increase HDL cholesterol and improve other markers of abnormal lipid metabolism in coronary artery disease patients with low initial HDL cholesterol levels. At weeks 6 and 12, HDL cholesterol was 12–14% and 14–16% higher, referring to Pakistani and American almonds, respectively.

Furthermore, a more recent meta-analysis [315] reported that avocado consumption significantly increased HDL cholesterol with significant heterogeneity. This remained consistent in sensitivity and subgroup analyses. Therefore, although HDL cholesterol has been declared for decades as an essential indicator to protect our health, in some contexts where different health factors are present, such as physical activity or healthy eating habits, the subtle reduction of this component might not be a problem because a whole-food plant-based diet is considered more protective. Finally, Table 2 displays a summary of findings on NCCD and MetS, while Figure 2 shows a brief description of PBD benefits for NCCD and MetS, and Figure 3 provides a comprehensive overview addressing the overall advantages of adopting a plant-based diet.

## 5. Conclusions

Over the past two decades, a substantial body of consistent evidence has emerged at the cellular and molecular level, elucidating the numerous benefits of a plant-based diet (PBD) for preventing and mitigating conditions such as atherosclerosis, chronic noncommunicable diseases, and metabolic syndrome. It is paramount to prioritize the consumption of quality, natural, and fermented foods to fully harness the health potential of this dietary approach. With guidance from qualified professionals to ensure optimal nutrition, any concerns regarding potential nutritional deficiencies can be effectively addressed through diverse and well-planned food choices. This specialist support enables individuals to adopt a PBD at any stage of life, allowing them to reap its benefits while minimizing potential risks. Consequently, a plant-based diet offers a promising outlook for improving the health and well-being of the global population, with its protective effects mediated by bioactive compounds. It is crucial for both the public and researchers to recognize the significance of this evidence and its implications for nutrition science and public health. As our understanding of the underlying mechanisms of a PBD continues to expand, there remain exciting areas within this field of study to explore and uncover.

## Figures and Tables

**Table 1:** Influence of certain plant components on markers linked to atherosclerosis.

Study	Analysis	Resume of Main Results Found										
		Apo-B	LDL-c	oxLDL	HDL-c	H/L-cr	TG	CVD/CHD	PROINF	SFA	UFA	FCF
[59]	EVOND vs. WD	x	↓	↓	x	x	↓	x	x	x	x	↓
[60]	HUF vs. HSF	x	↓	x	=	x	x	x	x	↓	↑	x
[61]	Reducing SFA intake	x	↓	x	x	x	x	x	x	x	x	x
[65]	SFA vs. RO (both rich fats)	x	↑	x	x	x	↑	↑	x	↑	x	x
[69]	Peanuts and walnuts consumption	x	x	x	x	x	x	↓	x	x	x	x
[71]	WW vs. WO	x	↓	x	x	x	↓	x	x	x	x	x
[76]	Changing SFA or CARB by UFA	x	↓	x	=	↑	↓	↓	x	x	x	x
[77]	LSAFA vs. HSAFA	=	=	x	↓	↓	↑	x	x	x	x	x
	HUFA vs. LSAFA or HSAFA	↓	↓	x	↓ or =	↑	↓	x	x	x	x	x
[79]	Chia vs. Control	x	=	x	↑	x	=	x	x	x	x	x
[104]	PBD w/EGGs vs. wo/EGGs	x	=	x	↑	=	=	x	x	x	x	x
[119]	High SFA diet vs. PUFA diet	↑	↑	x	↑	x	↑	x	↑	x	x	x
[120]	Walnuts replacing MUFAs	↓	↓	x	=	↑	=		↓	x	x	x
[121]	Walnuts-enriched diet vs. Control	x	=	x	=	x	=	x	x	x	x	x
[125]	Nitrate-rich beetroot juice	x	x	=	x	x	x	↓	=	x	x	x
[152]	Higher vs. Lower fiber intake	x	x	x	x	x	x	↓	x	x	x	x
[162]	Whole-grain cereal vs. Control	x	x	x	x	x	↓	x	x	x	x	x
[166]	Chungtaejeon (Fermented Tea)	x	x	x	x	x	x	↓	↓	x	x	x
[168]	β-glucans intake	x	↓	x	=	x	=	↓	↓	x	x	x
[174]	Berries consumption	=	↓	x	↑	x	↓	↓	↓	x	x	x
[177]	Mulberry leaf polyphenols effects	x	x	↓	x	x	x	x	↓	x	x	x

Apo-B: apolipoprotein B; LDL-c: low-density lipoprotein cholesterol; oxLDL: oxidized LDL; HDL-c: high-density lipoprotein cholesterol; H/L-cr: HDL/LDL cholesterol ratio; TG: triglycerides; CVD: cardiovascular disease; CHD: coronary heart disease; Proinf: proinflammatory cytokines; SFA: saturated fatty acids; UFA: unsaturated fatty acids; FCF: foam cell formation; EVOND: extra-virgin olive oil and nuts; WD: Western diet; HUF: high-unsaturated fat diet; HSF: high-saturated fat diet; RO: rapeseed oil; WW: whole walnuts; WO: walnuts oil; CARB: carbohydrate; LSAFA: low-saturated fatty acid diet; HSAFA: high-saturated fatty acid diet; HUFA: high-unsaturated fatty acids; PBD: plant-based diet; MUFAs: monounsaturated fatty acids. For more details see the main text. x: not measured or not informed; Arow down (↓): reduced parameter; Arrow up (↑): increased parameter; Equal sign (=): without change; Green box: positive change; Red box: negative change. Yellow box: no change.

**Table 2:** NCCD and MetS (pre-clinical, clinical, prospective, or follow-up human studies summary).

Study	Analysis	Resume of Main Results Found										
		IR/IS	DB *	GI	VF	TG	WC	BW	BMI	BP	HDL	LDL
[185]	Flavonoids	x	↑	x	x	x	x	x	x	x	x	x
[188]	Lifelong PBD adherence	x	↑	x	x	x	x	x	x	x	x	x
[191]	PBD	x	↑	x	x	x	x	x	x	x	x	x
[197]	Polyphenols	↑	↑	x	x	x	x	x	x	x	x	x
[202]	Genistein	↑	x	x	x	x	x	x	x	x	x	x
[203]	Flavan-3-ols and isoflavones	↑	x	x	x	x	x	x	x	x	x	x
[204]	Genistein	↑	↑	x	x	↓	x	x	x	x	x	x
[205]	PBD	x	↑	x	x	x	x	x	x	x	x	x
[206]	PBD	↑	↑	x	x	x	x	x	x	x	x	x
[209]	PBD	↑	x	x	x	x	x	x	x	x	x	x
[211]	PBD	x	x	x	x	x	x	x	x	↑	x	x
[212]	PBD (results observed only in males)	x	x	x	x	x	x	x	x	↑	x	x
[213,214]	PBD	x	x	x	x	x	x	x	x	↑	x	x
[215]	Diet change to PBD	↑	x	=	x	=	↓	↓		↑	↓	↓
[216]	Berries anthocyanin (women)	x	x	x	x	x	x	x	x	↑	x	x
[229]	Healthy PBD vs. Unhealthy PBD	x	x	x	x	x	x	x	x	↑	x	x
[232]	Vegan diet	x	x	x	x	x	x	x	x	x	x	↓
[234]	PBD	x	x	x	x	↓	x	x	x	x	x	↓
[244]	Vegans vs. Lacto-vegetarians	x	x	x	x	x	x	x	x	x	x	↓
[245]	Walnut’s intake (results men > women)	x	x	x	x	↓	x	x	x	x	x	
[248]	Quinoa bar consumption (30 days)	x	x	x	x	↓	x	x	x	x	x	↓
[250]	Blueberries	↑	x	x	x	x	x	x	x	x	x	x
[253]	Berberine (plant-derived compound)	↑	x	↑	x	x	x	x	x	x	x	x
[254,255,256]	Polyphenols	↑	x	↑	x	x	x	x	x	x	x	x
[257]	Strawberry and cranberry polyphenols	↑	x	x	x	x	x	x	x	x	x	x
[258]	Grape polyphenols	↑	x	x	x	x	x	x	x	x	x	x
[259]	Fruits and vegetables	x	x	x	↓	↓	x	x	x	x	x	x
[260]	Pesco-vegetarian and vegetarians	x	x	x	x	x	↑	↑	↑	x	x	x
[261]	PBD adherence	x	x	x	x	x	↑	x	↑	x	x	x
[262]	PBD adherence and high-flavonoid intake	x	x	x	x	x	x	x	x	↑	x	x
[263]		x	x	x	x	x	x	x	x	=	x	x
[264]		x	x	x	x	x	x	x	x	↑	x	x
[246]	PBD adherence	x	x	x	x	↑	x	x	x	x	↓	x
[265]	PBD adherence	x	x	x	x	↑	x	x	x	x	x	x
[266]	Vegetarians vs. omnivores	x	x	x	x	↓	x	x	x	↓	x	↓
[267]	PBD adherence	x	x	x	x	↓	x	x	x	x	↓	↓
[268]	Tomato	x	x	x	x	x	x	x	x	x	↑	x
[269]	Pakistani and American almonds	x	x	x	x	x	x	x	x	x	↑	x
[270]	Strawberry anthocyanin	↑	x	x	x	x	x	x	x	x	x	x

IR/IS: insulin resistance or insulin sensitivity; DB: diabetes or diabetic complications; GI: glucose intake; VF: visceral fat; TG: triglycerides; WC: waist circumference; BW: body weight; BMI: body mass index; BP: blood pressure; HDL: high-density lipoprotein; LDL: low-density lipoprotein; PBD: plant-based diet; NCCD: non-communicable chronic diseases. For more details see the main text. x: not measured or not informed; Arrow down (↓): decrease or negative association; Arrow up (↑): improvement or positive association; Equal sign (=): without change; Green box: positive change; Red box: negative change. Yellow box: no change. * Diabetes: type 1 or type 2 diabetes.

## Data Availability

Not applicable.

## Abbreviations

AMPK	AMP-activated protein kinase
DHA	docosahexaenoic acid
EPA	eicosapentaenoic acid
FMD	flow-mediated dilatation
HDL	high-density lipoprotein
LDL	low-density lipoprotein
MetS	metabolic syndrome
PBD	plant-based diet
PDI	plant-based diet index
PUFAs	polyunsaturated fatty acids
TMAO	trimethylamine N-oxide
SCFAs	short-chain fatty acids
TNF-α	tumor necrosis factor-alpha
ABC	ATP-binding cassette
ASCVD	atherosclerotic cardiovascular disease
